# Effectiveness of community-based folate-oriented tertiary interventions on incidence of fetus and birth defects: a protocol for a single-blind cluster randomized controlled trial

**DOI:** 10.1186/s12884-020-03154-w

**Published:** 2020-08-20

**Authors:** Mengru Li, Yi Zhang, Xiaotian Chen, Dingmei Wang, Mi Ji, Yuan Jiang, Yalan Dou, Xiaojing Ma, Wei Sheng, Weili Yan, Guoying Huang

**Affiliations:** 1 Pediatric Heart Center, Children’s Hospital of Fudan University, 399 Wan Yuan Road, Shanghai, 201102 People’s Republic of China; 2 Shanghai Key Lab of Birth Defect, Shanghai, China

**Keywords:** Serum folate, Red blood cell folate, Birth defects, Periconception health care, Cluster randomized controlled trial, Study protocol

## Abstract

**Background:**

Birth defects are the main cause of fetal death, infant mortality and morbidity worldwide. However, the etiology of birth defects remains largely unknown. Maternal folate status during periconception plays an important role in organogenesis and folic acid supplement reduces the risk of neural tube defects, congenital heart diseases, and several other birth defects. This trial seeks to evaluate the effectiveness of folate-oriented tertiary interventions during periconception on the incidence of fetus and birth defects.

**Methods:**

This is a single-blind, two-arm cluster randomized controlled trial in Shanghai, China. Eligible women from 22 clusters are recruited at pre-pregnancy physical examinations clinical settings. Compared to the routine perinatal care group (control arm), folate-oriented tertiary interventions will be provided to the intervention arm. The core interventions consist of assessments of folate status and metabolism, folate intake guidance, and re-evaluation of folate status to ensure red blood cell folate level above 400 ng/ml (906 nmol/L) before pregnancy. Screening and consulting of fetus and birth defects, and treatments of birth defects during pregnancy and afterward will be provided to both arms. The primary outcome is a composite incidence of fetus defects, stillbirth, and neonatal birth defects identified from the confirmation of pregnancy to 28 days after birth. Secondary outcomes include maternal and offspring adverse complications and cost-effectiveness of folate-oriented tertiary interventions. This protocol adheres to the SPIRIT Checklist.

**Discussion:**

To achieve the recommended folate status before or during pregnancy is still a challenge worldwide. This community-based cluster-randomized controlled intervention trial will evaluate the effectiveness of a package of interventions aiming at achieving recommended maternal folate status covering pre- and during pregnancy in reducing fetus and birth defects. Our study has the potential to improve the community-based practice of reducing modifiable risk factors of disease and improving primary prevention of the defects in China. The procedures would formulate the policy on folic acid supplementation during periconception against birth defects in primary care settings.

**Trial registration:**

Clinical Trial Registry, NCT03725878. Prospectively registered on 31 October 2018.

## Background

Birth defects, known as congenital anomalies or congenital malformations, are structural or functional anomalies that occur during intrauterine and can be identified before and after delivery [1]. Birth defects are a life-threatening cause leading to neonatal death, stillbirth, and long-term disability, which significantly influences individuals’ physical and mental health, health-care systems, families, and societies [2]. An estimated 303,000 newborns die within 4 weeks of birth every year caused by congenital anomalies [3]. Congenital heart defects (CHDs), oral clefts, hydrocephalus, neural tube defects (NTDs) and mental retardation are the most common birth defects in China. Genetic, infections, nutritional or environmental factors involved in the development of congenital anomalies. Among them, the role of folate in organogenesis and birth defects has been widely studied.

The protective effect of folic acid supplementations on several birth defects is well-established [4–6]. The efficacy of folic acid in preventing the occurrence and the recurrence of NTDs has been conclusively demonstrated through randomized controlled trials (RCTs) and has been confirmed in a public health campaign to ask women preparing for marriage taking a pill containing 400μg of folic acid till the end of their first trimester of pregnancy conducted between 1993 and 1995 in China [4, 5]. Red blood cell (RBC) folate concentrations above 400 ng/mL (906 nmol/L) has been recommended as the optimal cut-off level to achieve the greatest reduction of NTDs for women of reproductive age [7]. Populations based studies also showed strong evidence on folic acid supplementation reducing the occurrence of congenital heart disease (CHD) [8, 9]. And a reduction of CHD subtypes prevalence, such as conotruncal defects and coarctation of the aorta, was found being strongly related to periconceptional food fortification with folic acid [8]. The protective effect of folic acid supplementation reducing other outcomes has also been observed, such as cleft lip and palate, miscarriages or other birth defects [10–12]. However, the association between folic acid and the cleft lip was largely inconclusive for lacking RCTs evidences [13].

Insufficient folic acid intake and abnormal folate metabolism are the main determinants to folate status. Multiple single nucleotide polymorphisms (SNPs) in folate metabolism pathway genes are common causes of unclear low folate status in women who take sufficient folic acid. The C677T polymorphism on 5, 10-methylenetetrahydrofolate reductase (*MTHFR*) gene which encodes the pivotal metabolic enzyme can reduce the MTHFR enzyme activity and lower maternal folate status, and increase the susceptibility of NTDs and CHDs [14, 15]. Studies showed SNPs in folate metabolism pathway genes may increase the risk of CHDs via different mechanisms [16–19]. Therefore, it is necessary to consider folate metabolism ability in performing the individual folic acid supplementary guidance.

The importance of sufficient folate nutrition during the periconception period to fetal development is widely known and many countries have fortified foods with folic acid. However, more than half of women in those countries failed to achieve the optimal folate status at childbearing age [20], which may result from individual genetic variations. We believe that preconception-initiated folic acid supplementation to achieve the recommended folate levels before pregnancy, based on the current RBC folate status and the genetic background of women of reproductive age, will exert a strong effect on primary prevention of both fetus and birth defects. We designed a community-based cluster-randomized controlled intervention trial to evaluate the effectiveness of the procedures on the incidence of fetus and birth defects.

## Hypothesis and objectives of the study

### Hypothesis

We hypothesized that pre-pregnant women who receive folate-oriented tertiary interventions will have a significantly lower incidence of fetus and birth defects compared to those who receive the routine health care alone.

### Primary objective

To evaluate the effectiveness of community-based folate-oriented tertiary interventions focusing on achieving sufficient RBC folate levels before conception among pregnant-planning women on the incidence of fetus and birth defects.

### Secondary objectives

To determine the effectiveness of community-based folate-oriented tertiary interventions among pregnant-planning women in reducing the incidence of death or severe organ dysfunctions.

To analysis the cost-effectiveness of the tertiary intervention.

To determine the effect of community-based folate-oriented tertiary interventions with pregnant-planning women on the improvement of metabolic and development-related outcomes of mothers and children.

## Methods/design

### Study design

This is a single-blind two-arm cluster-randomized controlled trial (cRCT). A total of 22 community-based primary health care centers will be recruited as clusters in Minghang and Songjiang districts in Shanghai, China. Both of the two districts contain rural areas and urban areas, selected health care centers routinely provide service for over five million local residents (see Additional file 1: Table S1).

**Fig. 1:**
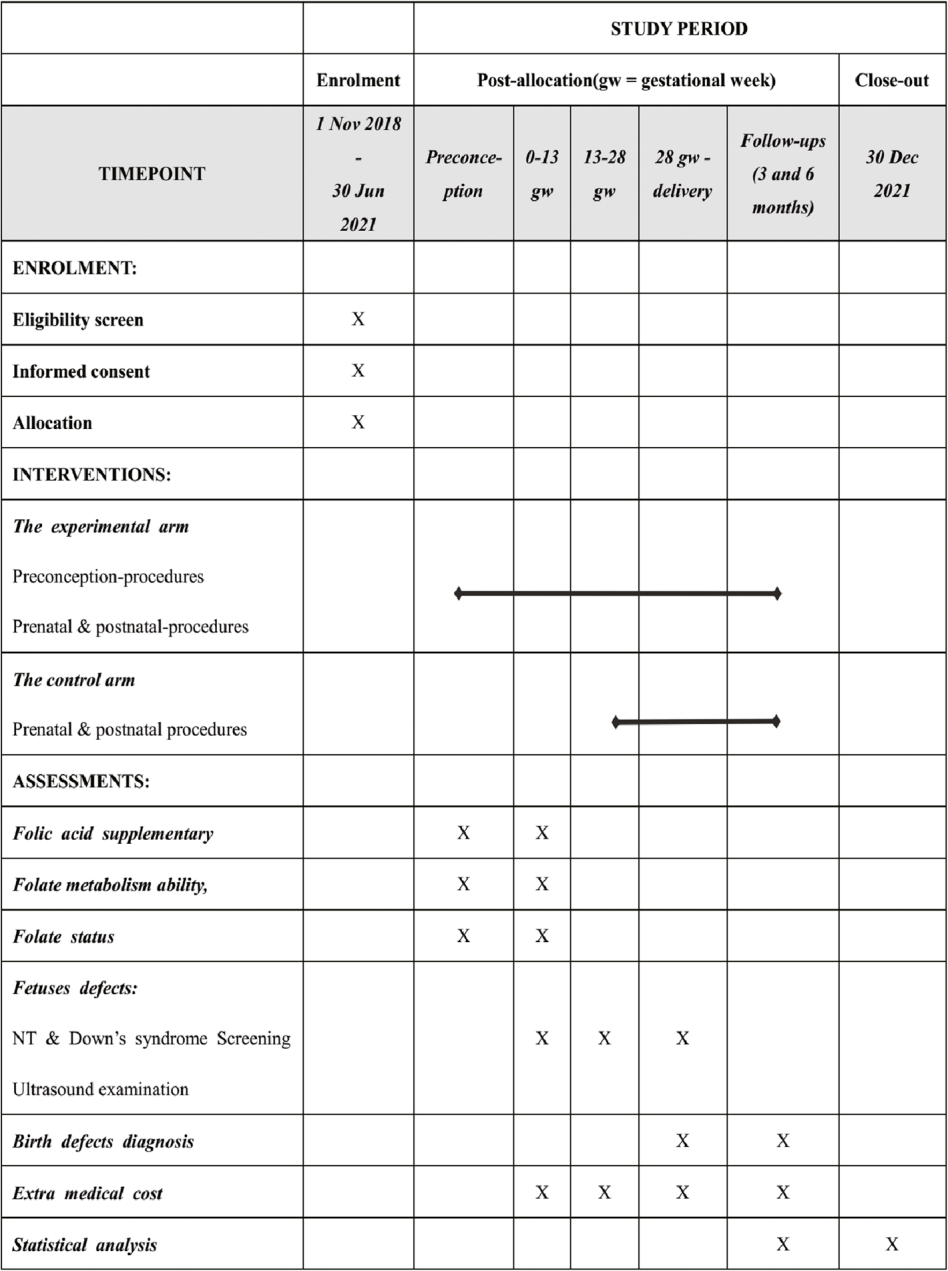
Flow diagram of enrolment, interventions, and assessments

**Fig. 2:**
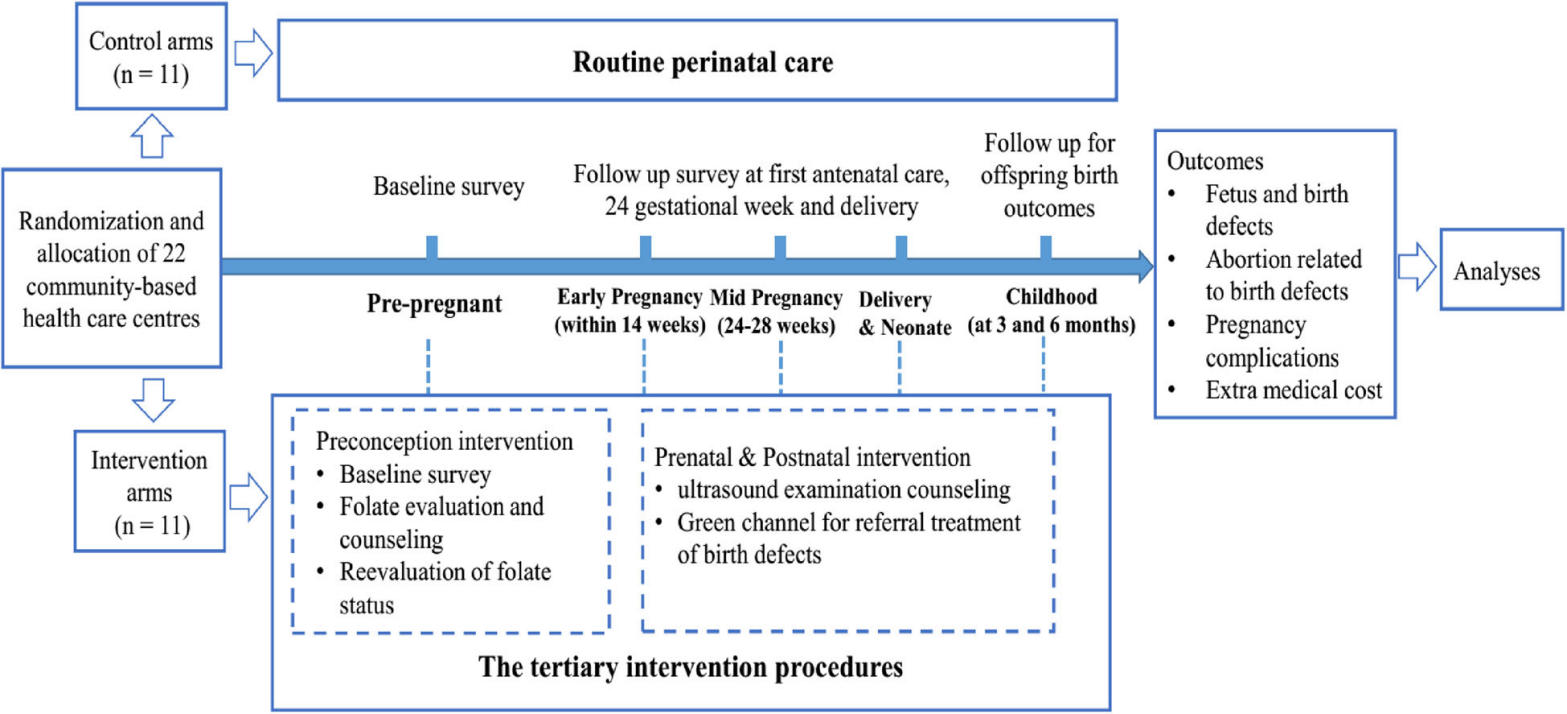
Flow diagram for the trial of the folate-oriented tertiary interventions on fetus and infant outcomes

This protocol refers to the SPIRIT 2013 Statement (Standard Protocol Items: Recommendations for Interventional Trials). A SPIRIT flow diagram see Fig. 1 and a SPIRIT checklist see Additional file 2: SPIRIT Checklist. The procedures of the trial is presented in Fig. 2.

### Participants and recruitment

The study population consists of women preparing for pregnancy. Participants meeting the following criteria are eligible to this study: (1) women planning for pregnant within a year; (2) women aging 18 to 45 years. (3) Females and their husbands attend premarital or pre-pregnancy physical examinations from Minhang and Songjiang districts in Shanghai. There are no exclusion criteria.

Eligible women are recruited at pre-pregnancy physical examinations clinics or premarital health checkup centers in Minhang and Songjiang districts in Shanghai. A written informed consent would be obtained from each participant during recruitment. The recruitment will be stopped until 2420 participants (> 110 pregnant women for each cluster) getting pregnant.

### Interventions

#### The control arm

The participants would only receive routine perinatal care according to the national guidelines [21], including premarital health checkups, pre-pregnancy physical examinations, antenatal cares, Nuchal translucency (NT) examinations (12-14th gestational week), Down’s screenings (14-20th gestational week), major structural malformations screened by ultrasound examination (20-24th gestational week), and neonatal health care.

#### The intervention arm

Participants in the intervention arm would be offered a package of folate-oriented tertiary interventions, including preconception, pregnant and neonatal procedures. The details are following as:

(i) Preconception procedures

The intervention arm will receive folate status evaluation by serum and RBC folate concentrations measurement. Combined the self-administered questionnaire and the folate metabolism ability (evaluated by genotyping key genetic variants), an individualized folic acid supplementary guide by phone counseling and a follow-up of folate status would be provided to whom with RBC folate below 400 ng/ml. The core rationale of preconception intervention following advises: (1) to take folic acid if one did not; (2) to take sufficient dose of folic acid if one did not; (3) to take sufficient dose of folic acid at least 3 months if one did not; (4) to take larger dose of folic acid if one with risk genetic variants; (5) to reevaluate RBC folate levels until one’s RBC folate levels achieve recommended levels before getting pregnant.

(ii) Prenatal procedures

Participants will be suggested to take folic acid until the end of the first trimester. Participants who will be screened with a fetus with defects will be referred to an authorized tertiary obstetric hospital and pediatric hospital, and receive prenatal consultations and treatments provided by a multi-disciplinary clinical team.

(iii) Postnatal procedures

The postnatal with defects will be transferred to Children’s Hospital of Fudan University to receive treatments and rehabilitation.

The referral procedures during prenatal and postnatal periods will also be provided to participants in the control arm considering ethics. This will attract more couples taking part in the study and stimulate participants’ adherence to the protocol.

### Outcomes

The primary outcome is defined as the occurrence of fetus defects, stillbirth, and neonatal birth defects identified from the confirmation of pregnancy to 28 days after birth. This composite outcome covered a total of 24 types of defects according to the national birth defects surveillance policy (Details of defects types see Additional file 3: Table S2). Besides, detailed information on both miscarriages and artificial abortions will be collected, including the time of abortion, reasons for abortion, and antenatal information before an abortion.

The secondary outcomes include: (1) the proportion (%) of total abortions that related to congenital defects. (Time Frame: from the confirmation of pregnant to the 28th gestational week); (2) the incidence (%) of infant death or severe organ dysfunctions (composite outcome) (Time Frame: From birth to 6 months after delivery (can be expanding to the end of the 7th month)); (3) extra medical cost that related to fetus and birth defects during pregnancy and after birth (Time Frame: from confirmation of pregnancy to one-year-old after birth).

### Sample size and power analysis

Sample size calculation is performed based on the primary outcome: the incidence of a composite rate of fetus defects, stillbirth, or birth defects. The proportion of primary endpoint was conservatively estimated to be 8% in the control arm according to two observational studies [22, 23], and 4% in the intervention arm (or 50% reduction) based on a meta-analysis of randomized intervention trials of folic acid supplementation intervention in the folate-deficient population [24]. With a cluster size of 100 subjects per cluster, K coefficient of 0.23, a power of 85% and a 5% level of type I error, 22 clusters (2200 pregnant women or 1100 in each arm) will be required. At least 110 pregnant women will be recruited in each cluster considering a 10% drop rate. Sample size and power analysis were performed by SAS® software version 9.4 (SAS Institute, Inc., Cary, NC, USA).

### Randomization and blinding

Permuted block randomization method with a block size of 4 will be used. Stata 15 was used for randomization code generation. Twenty-two community health care centres (clusters) will be allocated to the intervention and control arms. The randomization list will be generated by an independent statistician.

The study is single-blinded. The investigators who are involved in data collection, laboratory examination, and outcome assessments will be blinded. The statisticians will also be blinded when they perform analysis.

### Measurements

#### The nutrient supplement questionnaires

Two questionnaires will be used for nutrient supplementary data collection. Questionnaire one will be administered at recruitment before pregnancy to collect information on folic acid and vitamins supplement, the brand and content of nutrient supplements, maternal education, socio-demographic status, occupation, smoking status, alcohol consumption, and BMI. Questionnaire two will be used at early pregnancy. Besides the information in Questionnaire one, medications, reproductive history and health status will be included. The details of the information collected at each time-point are shown in Table 1.

**Table 1:** Summary of data collection and timeline

Data	Pre-pregnancy (couples)	Prenatal (female)			postnatal		
	Recruiments	First trimester	Second trimester	Third trimester	Neonatal	At 3 month	At 6 month
General characteristics							
Sociodemographic	**×**	**×**					
Nationality	**×**	**×**					
Educational level	**×**	**×**					
Occupation	**×**	**×**					
Incomes	**×**	**×**					
Weight	**×**	**×**	**×**	**×**			
Height	**×**	**×**					
Nutrients supplements							
Folic acid	**×**	**×**					
Multivitamin	**×**	**×**					
Single Vitamin	**×**	**×**					
Iron	**×**	**×**					
Calcium	**×**	**×**					
Zinc	**×**	**×**					
Smoking status	**×**	**×**					
Drinking status	**×**	**×**					
Last menstrual period time		**×**					
Reproductive history		**×**					
Chronic disease							
Diabetes		**×**					
Hypertension		**×**					
Family health history		**×**					
Herbal Drug		**×**					
Chemical exposure		**×**					
Fetus abnormality		**×**	**×**	**×**			
Drug use		**×**					
Infants							
Gender					**×**		
Mode of delivery					**×**		
Birth weight					**×**		
Length at birth					**×**		
Weight						**×**	**×**
Length/height						**×**	**×**
Head circumference					**×**	**×**	**×**
Waist circumference					**×**	**×**	**×**
Birth defects					**×**	**×**	**×**

#### Collection of blood samples

In this study, the remaining blood samples (serum and Ethylene Diamine Tetraacetic Acid (EDTA) anticoagulation) for routine clinical examinations will be collected and divided into light-proof tubes within 6 h and temporarily stored in a − 20 °C refrigerator at sites in both arms. Samples will be transported to the central laboratory for storage in microfuge by three trained investigators. Only samples in the intervention arm will be conducted the nutrient evaluation, and others will be stored for a future test.

#### Nutrient evaluation and genotyping of key enzymes

Only samples in the intervention arm will be conducted the nutrient evaluation, and others will be stored for a future test. EDTA anticoagulation blood will be used for measuring RBC folate and serum for folate, homocysteine, vitamin D, vitamin B12 and ferritin assays using an electrochemiluminescence assay (ARCHITECT i2000SR Analyzer, Abbott Laboratories, USA). RBC folate concentrations will be adjusted using hematocrit if the RBC folate concentration is below 150.0 ng/ml and the serum folate level is over 3.5 ng/ml. Examinations will be performed in the central laboratory of Children’s Hospital of Fudan University. The serum concentration of vitamin A and vitamin E will be quantitatively detected by liquid chromatography-tandem mass spectrometry (LC/MS/MS System, NO: API 3200MDTM, ABSciex Pte.Ltd.). Fasting serum cholesterol, high-density lipoprotein, low-density lipoprotein, triglyceride, and glucose levels will be measured using the Beckman Coulter AU chemistry analyzers (Beckman Coulter Inc., USA) in the central laboratory of the Children’s Hospital of Fudan University. Serum levels of Mg, Fe, Zn, Se, Mn, As, Cu, and Ca be analyzed by inductively coupled plasma mass spectrometry (iCAP6300, Thermo Scientific, USA) in the Instrumental Analysis Center of Shanghai Jiao Tong University.

The SNPs involved in folate metabolism pathway will be genotyped: *BHMT* (rs3733890), *CBS* (rs2850144, rs2851391), *FIGN* (rs2119289), *MTHFD1* (rs2236225), *MTHFR* (rs1801131, rs1801133, rs3737965), *MTR* (rs1805087, rs28372871, rs1131450), *MTRR* (rs1801394, rs326119), *RFC1* (rs1051266), and *SHMT* (rs1979277) [19, 25–29]. Genotyping will be performed using the TaqMan allelic discrimination assay in 96-well plates on the platform of 7500 Real-time polymerase chain reaction (PCR) System (Applied Biosystems, Foster City, CA). These biomarkers evaluated in this study are listed in Table 2.

**Table 2:** Parental biomarkers evaluated in the study

Biomarkers	Sample type	Baseline	During early pregnancy
		(Couples)	(Females)
Folate, ng/ mL	Serum	**×**	**×**
RBC Folate, ng/ mL	Whole blood	**×**	**×**
Homocysteine, μmol/L	Serum	**×**	**×**
Vitamin D, ng/mL	Serum	**×**	**×**
Vitamin B12, pg/mL	Serum	**×**	**×**
Vitamin A, μg/mL	Serum	**×**	**×**
Vitamin E, μg/mL	Serum	**×**	**×**
Iron protein, ng/mL	Serum	**×**	**×**
Metals (Fe, Zn, Se, Cu, Ca, etc), mg/L	Serum	**×**	**×**
DNA	Whole blood	**×**	
Genotyping	DNA	**×**	**×**
CHOL, HDL, LDL, TG, Fasting glucose	Serum	**×**	**×**

#### Data management and analyses

All data is managed centrally in the project management office. STATA 15.0 will be used for statistical analyses. An ID number will be used as the unique code to connect the various stages of the database. Data will be stored at the research team’s office. Daily data checking will be carried out by the research coordinator. A database will be built using Access 2013 (Microsoft office Professional Plus 2013); double entry and checking will be performed by an assigned data entry team. Only the data managers can access to the database by passwords and back up the database daily.

The findings of the trial will be reported according to the CONSORT guidelines for cRCT. An intention-to-treat (ITT) analysis will be used for the primary outcome. The primary analyses will be performed by generalized estimating equation (GEE) model with intervention as fixed effects, baseline measurements as covariates, and clusters and patients as random effects. GEE model will be performed to estimate risk ratio and 95% confidence intervals (CI), as well as a risk difference and 95%CI, by adjusting cluster with link of identity. The clinical and healthcare factors will be identified as the covariates since that would influence the probability of receiving intervention and outcomes according to previous studies and biological plausibility.

All secondary outcomes will be analyzed based on the intention-to-treat (ITT) population. Binary secondary outcomes will be analyzed with the same strategy and method as that used for the primary outcome. For the analysis of continuous secondary outcomes, a general linear model (GLM) model will be employed with treatment as a fixed effect and with normal distribution and identity link function. The mean difference of outcomes with 95%CI will be derived from the GLM model. For repeated measurement variables, such as infant weight at a different visit, or maternal body weight during gestation, will be analyzed using a mixed-effect linear regression model.

The cost will be collected in Chinese *yuan* (¥) and then converted to international dollars using purchasing power parities and gross domestic product (GDP) deflators. The estimates include five costs: folate evaluation, folic acid supplements, fetus and birth defects diagnosis, the treatment related to fetus and birth defects, and intervention procedures. Cost-effectiveness analyses will be conducted in agreement with the Consolidated Health Economic Evaluation Reporting Standards [30] and will be determined by relating the difference in the 5 costs between two arms. The further comprehensive cost-effectiveness analysis will be described in a separate SAP. All analyses will be described in detail in the finalized and signed a statistical analysis plan before unmasking the study (see Additional file 4: Statistics Analysis Plan).

#### Trial management

A research management team will be set up for this study to monitor the process and verify protocol compliance. The team is also responsible for data, biological samples, quality control, and participant management. All investigators responsible for recruiting has been trained before the project started and strictly follows the standard recruiting procedures and criteria. Blood samples will be transported using cold chain by trained carriers to control the quality. A pilot study was conducted before starting the trial to assess the feasibility and reliability of the procedures. Data correctness will be assessed and serious adverse events will be reported to the ethics committee of the particular center and Children’s Hospital of Fudan University, who has the authority to terminate the trial according to local laws or institutional regulations. The investigators should keep each care center, ethics committee, and the journal informed of any major protocol modifications.

## Discussion

It is a consensus that peri-conceptional folic acid supplementation can prevent fetus and birth defects, and many countries adopted different strategies and procedures to improve population folate nutrition levels during periconception period. However, few studies focused on achieving the optimal RBC folate status before pregnancy. The present study will evaluate the effectiveness of the package of interventions to a high risk group among the entire population based on the current individual folate examinations on the reduction of the incidence of fetus and birth defects. The used intervention procedures that were integrated into the community-based routine peri-conceptional primary care service will be generalizable and helpful for improving practice of primary prevention of the defects.

Our primary outcome may be influenced by several factors. Firstly, since some pregnancy is un-planned, participants in the intervention arm may fail to achieve the optimal folate level before pregnancy and may reduce the intervention effectiveness. Secondly, some participants receiving individual folate guidance may poorly comply with our suggestion of reevaluating folate status to ensure RBC folate concentrations > 906 nmol/L before they plan to get pregnant. This will reduce the intensity and effectiveness of interventions because our critical interventions focused on peri-conception. Considering these situations, we collect participants’ blood samples when they attend the first antenatal care at early pregnancy (within 14 weeks of the last menstrual period) for examination of folate levels. This will help us to obtain a RBC folate levels that are close to the critical window of defects occurrence, since RBC folate is stable and can reflect the folate status of the latest two-three months. Besides, for humanitarian reasons and maternity and child health, we provide interventions during pregnancy and after delivery to both groups, doing this may also reduce the chance of finding group difference in effectiveness of interventions. In the pre-conception recruitment, only participants who get pregnant during the study period will be valid study subjects and included for the outcome analysis. Since the low birth rate in Shanghai, we will expand recruitment to ensure enough sample size for the primary outcome.

The use of individual randomization would be impractical because participants tend to be contaminated and interfere with the interventions. Therefore, a cluster-randomized clinical trial study is performed. The trial is conducted based on community-based health care centers that provide routine primary maternity and child care service. This system will improve the compliance of participants because the general practitioner has close connections with the women from their community.

The intervention has several advantages over routine health care: (i) Pre-pregnancy interventions are an individualized, comprehensive intervention program that references the questionnaire, genes, and nutrient levels in the blood; (ii) This intervention study builds a complete health-care network from pre-pregnancy to childbirth, from community to hospital, which could be widely applied in the future; (iii) This community-based cRCT study could provide a good representation of the study population. The results of this trial will provide evidence on the prevention of fetus and birth defects in primary care settings.

## Trial status

The Protocol Version is 1.0. The trial is prospectively registered at ClinicalTrials.gov with identifier number NCT03725878 on Oct 31, 2018. Recruitment started on Nov 1, 2018, and the study is currently recruiting participants and collecting pregnancy associated information.

## Supplementary information

**Additional file 1 Table S1.** A list of the participating hospitals and community care center in Songjiang and Minghang district of Shanghai.

**Additional file 2.** SPIRIT Checklist.

**Additional file 3 Table S2.** Types of fetus defects and birth defects. Notes: Defects were detected by Down’s syndrome screening, NT examination and Ultrasound examination during the second trimester; and the number and type of birth defects after childbirth are diagnosed by professional clinical team.

**Additional file 4.** Statistical analysis plan (SAP).

## Data Availability

The data generated and/or during current study are not publicly available due to incomplete study, but are available from the corresponding author on reasonable request.

## Abbreviations

BMI: Body mass index
CHD: Congenital heart disease
GEE: Generalized estimating equation
GLM: General linear model
ITT: Intention-to-treat
MTHFR: The 5, 10-methylenetetrahydrofolate reductase
NT: Nuchal translucency
NTDs: Neural tube defects
PCR: Polymerase chain reaction
RBC: Red blood cell
RCTs: Randomized controlled trials
SNPs: Single nucleotide polymorphisms
