# Supplementary material for: Effectiveness of community-based folate-oriented tertiary interventions on incidence of fetus and birth defects: a protocol for a single-blind cluster randomized controlled trial
Source: BMC Pregnancy Childbirth. 2020 Aug 20;20:475. doi: 10.1186/s12884-020-03154-w (PMC7439679; doi:10.1186/s12884-020-03154-w)
Supplement: Supplementary file 1 — Additional file 1 Table S1. A list of the participating hospitals and community care center in Songjiang and Minghang district of Shanghai. [file 12884_2020_3154_MOESM1_ESM.docx]

Table S1. A list of the participating hospitals and community care center in Songjiang and Minghang district of Shanghai

| Number | The participating hospitals and community care center | Work |
| --- | --- | --- |
| H1 | Songjiang Maternal and Child Health Center | Recruitment/Follow up |
| H2 | Shanghai Minhang District Maternal and Child Health Care Hospital | Recruitment/Follow up |
| H3 | Wujing Hospital, Minhang District, Shanghai | Recruitment/Follow up |
| H4 | Songjiang Maternal and Child Health Hospital | Follow up |
| H5 | Sijing Hospital, Songjiang District, Shanghai | Follow up |
| H6 | Shanghai Songjiang District Central Hospital | Follow up |
| H7 | Minghang hospital, Fudan University | Follow up |
| H8 | The Fifth People's Hospital of Shanghai, Fudan University | Follow up |
| H9 | Renji Hospital | Follow up |
| H10 | Shanghai General Hospital | Follow up |
| C1 | Jiuting community health care center | Follow up |
| C2 | Fangsong community health care center | Follow up |
| C3 | Yongfeng community health care center | Follow up |
| C4 | Sheshan community health care center | Follow up |
| C5 | Yueyang community health care center | Follow up |
| C6 | Zhongshan community health care center | Follow up |
| C7 | Xinbang community health care center | Follow up |
| C8 | Xinqiao community health care center | Follow up |
| C9 | Sijing community health care center | Follow up |
| C10 | Yexie community health care center | Follow up |
| C11 | Dongjing community health care center | Follow up |
| C12 | Xiaokunshan community health care center | Follow up |
| C13 | Qibao community health care center | Follow up |
| C14 | Meilong community health care center | Follow up |
| C15 | Pujiang community health care center | Follow up |
| C16 | Hongqiao community health care center | Follow up |
| C17 | Zhuanqiao community health care center | Follow up |
| C18 | Jiangchuan community health care center | Follow up |
| C19 | Wujing community health care center | Follow up |
| C20 | Xinzhuang community health care center | Follow up |
| C21 | Gumei community health care center | Follow up |
| C22 | Maqiao community health care center | Follow up |
