# Supplementary material for: Effectiveness of community-based folate-oriented tertiary interventions on incidence of fetus and birth defects: a protocol for a single-blind cluster randomized controlled trial
Source: BMC Pregnancy Childbirth. 2020 Aug 20;20:475. doi: 10.1186/s12884-020-03154-w (PMC7439679; doi:10.1186/s12884-020-03154-w)
Supplement: Supplementary file 3 — Additional file 3 Table S2. Types of fetus defects and birth defects. Notes: Defects were detected by Down’s syndrome screening, NT examination and Ultrasound examination during the second trimester; and the number and type of birth defects after childbirth are diagnosed by professional clinical team. [file 12884_2020_3154_MOESM3_ESM.docx]

**Table S2 Types of fetus defects and birth defects**

| **Diagnosis** |
| --- |
| Anencephalus |
| Spina bifida |
| Encephalocele |
| Congenital Hydrocephalus |
| Cleft Palate |
| Cleft Lip |
| Cleft Lip with Cleft Palate |
| Microtia (including Anotia) |
| Deformity of external ear(s) (except Microtia and Anotia) |
| Esophageal atresia or stenosis |
| Anorectal atresia (including Congenital Anorectal Malformations) |
| Hypospadia |
| Ectopocystis |
| Pes Equinovarus |
| Polydactylism |
| Syndactylia |
| Limb shortening |
| Congenital Diaphragmatic Hernia |
| Pcromphalus |
| Celoschisis |
| Conjoined Twins |
| Trisomy 21 syndrome |
| Congenital heart disease |
| Others |
