# Supplementary material for: Effectiveness of community-based folate-oriented tertiary interventions on incidence of fetus and birth defects: a protocol for a single-blind cluster randomized controlled trial
Source: BMC Pregnancy Childbirth. 2020 Aug 20;20:475. doi: 10.1186/s12884-020-03154-w (PMC7439679; doi:10.1186/s12884-020-03154-w)
Supplement: Supplementary file 4 — Additional file 4. Statistical analysis plan (SAP). [file 12884_2020_3154_MOESM4_ESM.pdf]

Effectiveness of Folate-Oriented Tertiary Interventions on Birth Defects (FOID)

Statistical Analysis plan

The Data Management and Statistical Analysis Plan is directed to support the aims of the FOID Study

Version 1.0

October 29, 2018 Draft

January 02, 2019 Modified

Weili Yan

YI Zhang

Xiaotian Chen

Duolao Wang

TABLE OF CONTENTS

PAGE

ABBREVIATIONS 3

1. INTRODUCTION 3

2. STUDY OBJECTIVES AND OUTCOMES..3
   2.1. Study Objectives..3
      2.1.1. Primary Objective 4
      2.1.2 Secondary Objectives 4
   2.2. Outcomes 4
      2.2.1. Primary outcome 4
      2.2.2. Secondary outcomes 4
      2.2.3. Case ascertainment and case definitions 5

3. STUDY DESIGN 6
   3.1. Design 6
   3.2. Trial Sites..7
   3.3. Interventions 7
   3.4. Randomisation and blind 9
   3.5. Sample Size...12

4. ANALYSIS POPULATIONS 13
   4.1. Study population data sets 13
   4.2. Analysis Close Date 14
   4.3. Data download...11

5. STATISTICAL ANALYSES 11
   5.1. Primary Outcome Analysis...11
   5.2. Secondary Outcome Analysis 13

6. GENERAL CONSIDERATIONS FOR DATA ANALYSE .... 错误!未定义书签。4
   6.1. Data Summaries...15
   6.2. Graphical Displays 16

1. INTRODUCTION

The purpose of this Statistical Analysis Plan (SAP) is to define the outcome variables, statistical methods, and analysis strategies to address the objectives of the single-blind cluster randomized controlled trial. The purpose of this project is to assess the effectiveness of a comprehensive tertiary interventions (before pregnancy, during pregnancy and after delivery) on the incidence and the clinical outcomes of fetus and birth defects in a preparing-for-pregnancy population in Shanghai. The preconception intervention is identifying individuals with red blood cell folate concentration below 400 ng/ml, a concentration recommended by World health organization (WHO) to prevent neural tube defects (NTDs). Then modifying their folate deficiency status to normal before pregnancy by individualized instruction for folic acid supplementary. A referral to a tertiary hospital to receive genetic assessments and prenatal diagnosis will be given to fetus probably with birth defect(s) during pregnancy. After delivery, newborns having birth defect(s) will receive a referral to professional clinical team and a 6-month follow up. The current project will provide evidence on the necessity and effectiveness of preconception intervention focusing on sufficient folate nutrition levels in prevention of birth defects.

2. STUDY OBJECTIVES AND OUTCOMES

2.1. Study Objectives

2.1.1. Primary Objective

The primary objective is to evaluate the effectiveness of the monitoring and endurance of sufficient folic acid supplement before conception on incidence of fetus and birth defects.

2.1.2 Secondary Objectives

The secondary objectives include: (1) to assess the incidence rate of total abortions (including miscarriages and artificial abortions) and stillbirths related to all kinds of congenital defects compared with control group; (2) to assess the incidence of infant death or severe organ dysfunctions compared with control group; and (3) to evaluate the medical costs related to fetus and birth defects during pregnancy and after birth compared with control group.

2.2. Outcomes

2.2.1. Primary outcome

The primary endpoint is defined as occurrence of fetus, still birth,

or neonatal birth defects identified from the confirmation of pregnancy to 28 days after birth. This is a composite outcome: detection of fetus defects detected by Down's syndrome screenings, neuro-tube defect (NT) examinations and ultrasound image examinations during the second trimester, stillbirth, and the number of birth defects after delivery diagnosed by clinical team (for the details of defects types see Appendix 1).

2.2.2. Secondary outcomes

- The occurrence of abortion that related with congenital defects. (Time Frame: from the confirmation of pregnancy to the 28th gestational week) (Binary-Yes/No)
- The occurrence of infant death or severe organ dysfunctions (composite outcome) (Time Frame: from birth to 6 months after delivery (can be expanding to the end of the 7th month)) (Binary-Yes/No).
- Medical cost that relates with fetus and birth defects during pregnancy and after birth (Time Frame: from confirmation of pregnancy to one year old after birth) (Continuous)
- Occurrence of confirmed congenital heart defects, via ultrasound screening fetus and neonatal screen confirmed by ECG (%) (Binary-Yes/No).
- Occurrence of maternal gestational diabetes, which is diagnosed by

routine oral glucose test at gestational weeks 24 (Binary-Yes/No).

- Maternal gestational weight gain (kg), which is calculated with body weight at delivery minus weight before pregnant (self-report) (Continuous).
- Maternal weight gain (kg) at early gestation (body weight at around weeks 20 minus preconceptional weight) (Continuous)
- Gestational hypertension (Binary-Yes/No)
- Maternal body weight (kg) at 6 month after delivery (Continuous)
- Maternal HbA1c (%) at 6 month after delivery (Continuous)
- Infant body weight (g) at birth, 42 day, 3 months, and 6 months (Continuous, repeated measurements).

2.2.3. Case ascertainment and case definitions

(1) Fetus and Birth defects

Defects identified from the 12 gestational weeks to 28 days after birth are included. In consistent with the national birth defects surveillance policy, a total of 24 types of defects are defined (details of types of defects types see Appendix 1). Among them, Down's syndrome, neural tube defects, congenital heart defects, hydrocephalus, digestive tract malformations and urinary malformations are most common defects in China. Defects will be detected by prenatal Down's syndrome screening, NT examination and ultrasound image examination during the second trimester; and the

number and type of birth defects after childbirth will be diagnosed by professional clinical team.

Down syndrome (DS), caused by the trisomy, translocation, or partial trisomy of chromosome 21, is the most common genetic cause of intellectual disability. DS is diagnosed by neonatologist. Neural tube defects including spina bifida and hypospadias, will be diagnosed by ultrasound examination and pediatric neurosurgeon. Congenital heart disease will be diagnosed by doppler ultrasound and CHD screening (pulse oximetry plus cardiac murmurs). Hydrocephalus, digestive tract malformations, urinary malformations and other defects also will also be diagnosed either by ultrasound or some other specific diagnosis methods.

(2) Abortion

Abortion is defined as pregnancy suspension including miscarriage and artificial abortion. Detailed information for abortions will be collected, including time of abortion, reasons for abortion especially and antenatal information before abortion.

(3) Pregnancy complications

Pregnancy complications are a composite of gestational severe adverse events including maternal problems and fetal and placental problems. Maternal problems defined in this study include gestational diabetes, hyperemesis gravidarum, pelvic girdle pain, high blood pressure, deep vein thrombosis, anemia, infection, peripartum cardiomyopathy and

hypothyroidism; Fetal and placental problems are defined as ectopic pregnancy, miscarriage, placental abruption, placenta praevia, placenta accrete, multiple pregnancies, vertically transmitted infection, intrauterine bleeding.

(4) Medical cost

The study will collect the cost of the intervention and treatment related with birth defects, and compared the medical cost in two group. The cost of intervention will include folic acid supplements, nutrients examinations before conception and the cost of fetus and birth defects screening. The cost of treatments related with birth defects will include treatment, nursing, and rehabilitation, which will be calculated according to the Shanghai medical routine fee standards. All the cost is settled in RMB.

3. STUDY DESIGN

3.1. Design

This project is a single blind cluster randomized controlled trial to assess the effectiveness of a package of comprehensive tertiary interventions before and during gestation in reducing the incidence of fetus defects, still birth or birth defects compared with the standard perinatal care. We will

recruit women in the preconception clinical visit. Among the enrolled participants, once a woman is confirmed for pregnancy, she will be followed throughout the entire pregnancy, and their babies will be followed up till 6 months old.

3.2. Trial Sites

The trial initially will recruit 22 community-based health care centers in Shanghai: Jiuting, Fangsong, Yongfeng, Sheshan, Yueyang, Zhongshan, Xinbang, Xinqiao, Sijing, Yexie, Dongjing, Xiaokunshan, Qibao, Meilong, Pujiang, Hongqiao, Zhuanqiao, Jiangchuan, Wujing, Xinzhuang, Gumei and Maqiao community.

3.3. Interventions

3.3.1 Intervention arms:

Standard tertiary interventions of birth defects:

Couples eligible for reproductive policy are entitled to routine health cares including general health cares (health education, medical history inquiry, physical examinations, consulting guidance and pregnancy outcome follow-up) and medical examinations (laboratory examinations, virus screenings and image examinations). But nutrients status is not included in these examinations, such as folate, vitamin B12 and

acroelements, etc. Regular antenatal cares are required, such as deformity screening by ultrasound. Routine neonatal screenings are conducted to diagnose infant with birth defect(s) timely.

Additional preconception health care -- identifying individuals with deficient RBC folate concentrations and providing individualized guide for folic acid supplementary:

Various studies have revealed that folate is essential in early embryo development, whereas folate level examination is not included in routine health cares among periconception women. Therefore, we examine subject's serum and red blood cell (RBC) folate concentrations to identify subjects with folate deficiency. By reviewing of subjects' folate supplement status from the subject-administered questionnaire, RBC folate concentration and folate metabolism ability evaluated by genotyping key genomic variants, an individualized folic acid supplementary guide is provided to these participants. The goal is to ensure their RBC folate concentration >400 ng/ml during periconception by individualized counselling.

Additional health care procedures during and after pregnancy -- Green channels for antenatal care and postnatal care:

In the both arm, fetus and infants with birth defect(s) will be referred

via a green channel to an authorized tertiary obstetric hospital and pediatric hospital respectively to receive further routine comprehensive assessments for prenatal diagnosis. The parents are referred to a multi-discipline pediatrics team for a sufficient consultation about possible postnatal treatments in the future if the pregnancy remains to avoid unnecessary abortions. All pregnancies will be followed up at delivery to help clinical team for early diagnosis, treatment and related procedures. All infants from the two arms will continuously be followed up till 6 months after birth to collect data of clinical outcomes.

The intervention includes standard tertiary interventions of birth defects (existing), plus preconception folate-oriented health care procedures; and additional health care procedures during and after pregnancy

3.4. Randomisation and blind

Permuted block randomisation method with blocksize of 4 will be used. Stata 15 was used for randomisation code generation. Twenty-two community health care centers (clusters) will be allocated to the intervention and control arms. Randomisation list will be generated by an independent statistician.

The study is single blinded, in which the participants will not know which arm they belong to. The study investigators who are involved in

recruitment, the laboratory staff, and the dose-adjusting investigator (who was not involved in participant care) will remain blinded. The investigator involved in intervention and the statistician was not blinded, as unbinding was required in order to carry out statistical procedures.

All researchers conducting outcome assessments will be masked in the trial. The trial statistician will also be blinded regarding the treatment code when he develops the statistical analysis plan and writes the statistical programmes, which will be validated and completed using dummy randomisation codes. The actual allocation will only be provided to the study team after lock of the database.

3.5. Sample Size

Sample size calculation was performed based on the primary outcome-the incidence of a composite rate of fetus, still birth, or birth defects. The proportion of primary endpoint was conservatively estimated to be 8% in the control arm according two observational studies (1, 2) and 4% in the intervention arm (or 50% reduction) based on meta-analysis of randomized intervention trials of folic acid supplementation intervention in folate-deficient population (3). With cluster size of 100 subjects per center, K coefficient of 0.23, a power of 85 % and a 5 % level of type I error, 22 clusters (2200 patients or 1100 in each arm) will be required. To allow for a 10 % dropout rate, at least 110 subjects will be recruited in each center.

4. ANALYSIS POPULATIONS

4.1. Study population data sets

Two study populations will be considered in the analysis as follows:

Intent-to-Treat population

Intent-to-treat (ITT) population will be defined as the subjects that are recruited from the randomized clusters before pregnancy and finally get pregnant. For the ITT analysis in this trial, participants will be recruited before pregnancy, only those who become pregnant will be followed up for outcomes. For example, if a participant does not respond to investigators' interventions, or subjects lost for follow-up for outcomes, or receives wrong treatment, or subjects in the control arm demand additional information and medical assistance from investigators), they will be included in the ITT population.

Participants will be **excluded from the ITT analysis if** the primary outcome of the study is missing, forming a modified ITT population (mITT).

Per-protocol population

Per protocol population will be defined after excluding participants who meet the following conditions:

Participants will be **excluded from the per-protocol population if they**:

- Do not respond to the allocated intervention;
- Switch intervention

The treatment groups in the per-protocol analysis will be defined according to what the participant will actually receive. This population will be used for the supportive analyses.

4.2. Analysis Close Date

The analysis close date is the date on which the last participant completed follow-up to achieve the primary outcome and until age of six months.

4.3. Data cleaning

The data will then be checked to ensure that there are no erroneous entries and that all missing data is properly coded. Any changes will be made on the ACCESS database.

4.4. Data download

For each time point, once all data have been inputted and checked, the database will be locked and a data download request made. The data will be downloaded into SAS, SPSS and STATA formats for statistical analyses.

5. STATISTICAL ANALYSES

5.1. Primary Outcome Analysis

5.1.1. ITT analysis of the primary outcome - the primary analysis

The primary outcome is a binary composite outcome: occurrence of stillbirth, fetal, or birth defects identified from the 12 gestational week until 28 days after birth. The primary analysis will be based on the ITT population as defined above.

A formal statistical analysis will be performed using generalised estimating equation (GEE) model with treatment as the only predictor and center as cluster effect. Binomial distribution and log link function will be used in the GEE model, from which the treatment effect will be estimated as the risk ratio between intervention and the control arm together with 95%CIs.

If the above log-binomial regression model does not converge, GEE model with normal distribution and log link function will be used.

5.1.2. Covariate adjusted analysis of the primary outcome

An analysis of the primary endpoint will be adjusted for age, BMI (body mass index), and adverse pregnant history (previous miscarriage), and exposure to smoking (mother or father's smoking status, yes/no) at baseline. GEE model will be performed to estimate risk ratio and 95%CI.

The above log-binomial GEE model may not converge when all covariates are introduced into the model simultaneously. If this occurs, a covariate will be removed from log-binomial GEE model one by one from the last covariate until GEE model converges.

5.1.3. Subgroup analysis of the primary outcome

Subgroup analyses will be performed on age, BMI, adverse pregnant history, exposure to smoking, RBC folate level at baseline. Age will be categorised at <35 or more; BMI will be dichotomized at 24kg/m2; RBC folate level will be cut off at 400 ng/ml before pregnancy.

Assessment of the homogeneity of treatment effect by a subgroup variable will be conducted by a GEE with the treatment, subgroup variable, and their interaction term as predictors, center as cluster, and the P-value presented for the interaction term. In addition, the risk ratio of the primary endpoint will be derived from the GEE model with binomial distribution function, and log link function, respectively.

5.2. Secondary Outcome Analysis

All secondary outcomes will be analysed as a superiority design and two-sided 95% CIs for the treatment differences in these outcomes between the two arms will be calculated and presented. Secondary outcome analyses will be based on the ITT population unless specified.

Analysis of binary outcomes

Binary secondary outcomes will be analyzed with the same strategy and method with that is used for the primary outcome. Generalised estimating equation (GEE) model will be performed with treatment as the only predictor and center as cluster effect using binomial distribution and log link function, from which the treatment effect will be estimated as the risk ratio between intervention and the control arm together with 95%CIs. If the above log-binomial regression model does not converge, GEE model with normal distribution and log link function will be used.

Analysis of continuous outcomes

The continuous outcome will be summarised using number of subjects (n), mean, standard deviation (SD), minimum, and maximum by intervention group, and will be analysed by a GLM model with treatment as fixed effect and with normal distribution and identity link function. Difference in mean outcome with their two-sided 95% confidence intervals between two groups will be derived from the GLM model.

For repeated measurement variables, such as baby body weight at different visit, or maternal body weight during gestation, will be analyzed using mixed effect linear regression model, with treatment, visit, interaction between treatment and visit as fixed effects, baseline measurement as covariate, cluster and subject as random effects.

Cost-effectiveness analysis

We calculate the total cost of each subject including cost of treatment, nursing, and rehabilitation from recruitment to the follow-up endpoint. The differences of total cost will be compared between the two arms using the same methods described above by treating which as a continuous variable. The total cost of intervention arm included folic acid supplements and RBC folate examinations before conception. All the cost is settled in RMB. Further comprehensive cost-effectiveness analysis will be described by a separated SAP.

6. GENERAL CONSIDERATIONS FOR DATA ANALYSES

STATA® (version 15.0) will be used to perform all data analyses and generate the majority of data displays. SAS or SPSS or S-Plus or R may also be used for some data analyses and generating statistical graphs.

6.1 Data Summaries

Continuous variables will be summarized according to number of subjects with non-missing data (n), mean, standard deviation (SD), median, minimum, and maximum. The confidence intervals will be reported on summaries of continuous effectiveness variables.

Categorical variables will be summarized according to the absolute frequency and percentage of subjects (%) in each category level. The

denominator for the percentages is the number of subjects in the treatment arm with data available, unless noted otherwise.

6.2 Graphical Displays

Mean values for some continuous outcomes by treatment and visit will be plotted.

ECT Study variable list

Table 1 Intervention trail variable list

NO	**variable**	
	**Pre-pregnancy**	
1	hospital card number	T
2	Recruited number	T
3	Recruited date	D
4	Female name	T
5	Female height	N
6	Female weight	N
7	Female age	N
8	Male name	T
9	Male height	N
10	Male weight	N
11	Male age	N
12	Tel No	T
13	Community (basis for grouping)	N
14	Nutrient Interventions	B (1=intervention 0=control)
	**Nutrient first test**	
15	Nutrient first test date	D
16	Serum folate	N
17	Red blood cell folate	N
18	Serum ferritin	N
19	VD(Vitamin D)	N
20	HCY(homocysteine)	N
21	Vitamin B12	N
22	LDL low density lipoprotein cholesterol	N
23	HDL high density lipoprotein cholesterol	N
24	TG total cholesterol	N
25	TC triglyceride	N
26	FBG(fasting blood-glucose)	N
27	Genotyping (BHMT (rs3733890), CBS (rs2850144, rs2851391), FIGN (rs2119289), MTHFD1 (rs2236225), MTHFR (rs1801131, rs1801133, rs3737965), MRT (rs1805087, rs28372871, rs1131450), MTRR (rs1801394, rs326119), RFC1 (rs1051266), and SHMT (rs1979277))	B
28	Clinical information (see Table 3)	-
29	Insufficient serum folate	B
30	Insufficient RBC folate	B

	**Nutrient repetition measurement**	
29	Nutrient repetition measurement date	D
30	Serum folate	N
31	Red blood cell folate	N
32	HCY (Homocysteine)	N
	**Pregnancy**	
33	Ultrasound image screen in mid-gestation	T
34	Report detail (positive)	B
35	Confirm image diagnosis	T
36	Therapeutic plan	T
37	birth defect diagnosis	T
38	Clinical information (see Table 2)	-

Note: T, text variable; D, The date type; N, Continuous variable; B, binary variable.

Table 2 Pregnant variable list in routine system

NO	variable	
	**Basic information**	
1	hospital card number	T
2	inpationt number	T
3	name	T
4	age	N
5	Pregnant times	N
6	Delivery times	N
7	last menstrual period	D
8	Gestational week at the first visit	N
9	The first visit date	D
10	Height	N
11	Weight	N
12	Systolic blood pressure at the first visit	N
13	Diastolic blood pressure at the first visit	N
14	Occupation	T
15	Education	T
	**Antenatal care record**	
16	Weight at eath antenatal care	N
17	Systolic blood pressure at eath antenatal care	N
18	Diastolic blood pressure at eath antenatal care	N
19	Gestational week at eath antenatal care	N
20	Antenatal care date	D
	**Lab data**	
21	Cytomegalovirus	N
22	Cytomegalovirus date	D
23	Rubella virus	N
24	Rubella virus date	D
25	Toxoplasmosis	N
26	Toxoplasmosis date	D
27	Syphilis screening	N
28	Syphilis screening date	D
29	Fasting blood-glucose	N
30	Fasting blood-glucose date	D
31	HCT(hematokrit)	N
32	HCT(hematokrit) date	D
33	Serum folate	N
34	Serum folate date	D
35	HCY(homocysteine)	N
36	HCY(homocysteine) date	D

37	OGTT 0 hours	N
38	OGTT 1 hours	N
39	OGTT 2 hours	N
40	OGTT date	D
41	Triglyceride	N
42	Triglyceride date	D
43	Total cholesterol	N
44	Total cholesterol date	D
45	Hemoglobin date	N
46	Hemoglobin date	D
	**Delivery date**	
47	Gestational week at delivery	N
48	Delivery mode	T
49	Birth weight	N
50	Birth weight(second baby)	N
51	Systolic blood pressure at delivery	N
52	Diastolic blood pressure at delivery	N
53	Apgar scoring	N
54	Delivery date	D
55	Birth defect records	T
56	Weight blood pressure at delivery	N

Note: The data will be extracted from maternal clinic antenatal medical record system. T, text variable; D, The date type; N, Continuous variable; B, binary variable.

Table 3 Pre-pregnant variable list in routine system

	variable	
	**Basic information**	
1	Wife id	T
2	Husband nation	T
3	Husband age	N
4	Husband education	T
5	Husband id	T
6	Husband occupation	T
7	Wife nation	T
8	Wife age	N
9	Wife education	T
10	Wife occupation	T
11	Tel no	T
12	Mobile phone No	T
	**Medical history**	
13	Female anemia	B
14	Female EH	B
15	Female heart disease	B
16	Female DM	B
17	Female epilepsia	B
18	Female thyroid disease	B
19	Female CGN	B
20	Female mental disease	B
21	Female tumour	B
22	Female TB	B
23	Female HBV	B
24	Female VD	B
25	Male anemia	B
26	Male EH	B
27	Male heart disease	B
28	Male DM	B
29	Male epilepsia	B
30	Male thyroid disease	B
31	Male CGN	B
32	Male mental disease	B
33	Male tumour	B
34	Male TB	B
35	Male HBV	B
36	Male VD	B
	**Vaccine**	
37	Female rubella vaccine	B
38	Female hepB vaccine	B

39	Male hepB vaccine	B
	**Durg**	
40	Female current medicine	B
41	Female medicine name	B
42	Male current medicine	B
43	Male medicine name	B
	**Childbearing history**	
44	Birth history	B
45	Pregnancy times	B
46	Live birth	B
47	Dead fetus	B
48	Dead birth	B
49	Term delivery	B
50	Premature delivery	B
51	Natural abortion	B
52	Abactio	B
53	Children number	B
54	Birth defect	B
55	Defect type	B
56	Menarche age	B
57	Period menstruation	B
58	Menstrual cycle	B
59	Menstrual quantity	B
60	LMP	D
	**Family history of disease**	
61	Female family history thalassemia	B
62	Female family history albinism	B
63	Female family history favism	B
64	Female family history hemophilia	B
65	Female family history CHD	B
66	Female family history DS	B
67	Female family history openNTDs	B
68	Female family history DM	B
69	Female family history dysnoesia	B
70	Female family history daysaudia	B
71	Female family history viaual disorder	B
72	Female family history neuropsychiatric	B
73	Female family history other birthdefects	B
74	Female family history fetal death	B
75	Female family history intermarry	B
76	Female family history relations	B
77	Male family history thalassemia	B
78	Male family history albinism	B

79	Male family history favism	B
80	Male family history hemophilia	B
81	Male family history CHD	B
82	Male family history DS	B
83	Male family history openNTDs	B
84	Male family history DM	B
85	Male family history dysnoesia	B
86	Male family history dysaudia	B
87	Male family history viaual disorder	B
88	Male family history neuropsychiatric	B
89	Male family history other birth defects	B
90	Male family history fetal death	B
91	Male family history intermarry	B
92	Male family history relations	B
	**Anthroposomatology**	
93	Female height	N
94	Female weight	N
95	Female BMI	N
96	Female heart rate	N
97	Female SBP	N
98	Female SDP	N
99	Male height	N
100	Male weight	N
101	Male BMI	N
102	Male heart rate	N
103	Male SBP	N
104	Male SDP	N
	**Lab data**	
105	Leucorrhea check	N
106	Clue cell	N
107	Monilia infection	N
108	Trichomomas	N
109	Cleanness	N
110	Whiff test	N
111	PH	N
112	Wom blood analysis	N
113	Female hb	N
114	Female wbc	N
115	Female rbc	N
116	Wom urine test	N
117	Female ABO	N
118	Female Rh	N
119	Female GLU	N

120	Female GLU levels	N
121	Female NG	N
122	Female chlamydia	N
123	Female syphilis	N
124	Female HIV	N
125	Female ALT	N
126	Female ALT levels	N
127	Female HBs-Ag	N
128	Female HBs-Ab	N
129	Female HBe-Ag	N
130	Female HBe-Ab	N
131	Female HBc-Ab	N
132	Female HCV-Ab	N
133	Female CMV IgM	N
134	Female CMV IgG	N
135	Female RV IgM	N
136	Female RV IgG	N
137	Female TOX IgM	N
138	Female TOX IgG	N
139	Male blood analysis	N
140	Male hb	N
141	Male wbc	N
142	Male rbc	N

Note: The data will be extracted the preconception care electronic data system T, text variable; D, The date type; N, Continuous variable; B, binary variable.

Appendix 1

Types of fetus defects and birth defects

1. Anencephalus
2. Spina bifida
3. Encephalocele
4. Congenital Hydrocephalus
5. Cleft Palate
6. Cleft Lip
7. Cleft Lip with Cleft Palate
8. Microtia (including Anotia)
9. Deformity of external ear(s) (except Microtia and Anotia)
10. Esophageal atresia or stenosis
11. Anorectal atresia (including Congenital Anorectal Malformations)
12. Hypospadia
13. Ectopocystis
14. Pes Equinovarus
15. Polydactylism
16. Syndactylia
17. Limb shortening
18. Congenital Diaphragmatic Hernia
19. Pcromphalus
20. Celoschisis
21. Conjoined Twins
22. Trisomy 21 syndrome
23. Congenital heart disease
24. Others.

REFFERENCE

1. Xuelei Yin XD, Mingzhu Yao. Analysis on the composition of 481 neonates with congenital malformation and their associated factors. j Clin Pediatr. 2008;26(3):204-8.

2. Lixin Zheng ML, Qiling Wang, Shanshan Xu, Ganhua Pan, Jiangchu Huang, Meizhen Zeng. Investigation on birth defects of fetus after twelve weeks pregnancy and chiIdren with 0-5 yares old in part area of Guangdong prevince chin J Fam Plann. 2017;25(2):93-7.

3. Blencowe H, Cousens S, Modell B, Lawn J. Folic acid to reduce neonatal mortality from neural tube disorders. Int J Epidemiol. 2010;39 Suppl 1:i110-21. doi:10.1093/ije/dyq028
